# Malignant Hypercalcemia Induced by the Ectopic Production of Intact Parathyroid Hormone (PTH)

**DOI:** 10.7759/cureus.34770

**Published:** 2023-02-08

**Authors:** Ana Neves, Inês Mendonça, José Alberto Cunha Marques, José Costa, Jorge S Almeida

**Affiliations:** 1 Internal Medicine, Centro Hospitalar Universitário de São João, Porto, PRT; 2 Intensive Care Medicine, Centro Hospitalar Universitário de São João, Porto, PRT; 3 Internal Medicine, Hospital Terras do Infante, Lagos, PRT; 4 Medicine, Faculdade de Medicina da Universidade do Porto, Porto, PRT

**Keywords:** small cell lung cancer, intact parathyroid hormone, ectopic malignant hyperparathyroidism, ectopic pth production, malignant hypercalcemia

## Abstract

Malignant hypercalcemia is a common finding in patients with advanced cancer, involving mechanisms like tumor secretion of parathyroid hormone (PTH)-related protein, osteolytic metastases, and tumor production of calcitriol. Although rare, hypercalcemia induced by ectopic tumoral secretion of PTH can be an additional mechanism. Here we present an 84-year-old male patient who was admitted to the hospital with a non-productive cough, anorexia, and a single episode of small-volume hemoptysis. He was diagnosed with stage T4N3M1c left lung small cell carcinoma, and laboratory tests were remarkable for elevated ionized calcium as well as elevated serum intact PTH. A parathyroid 99mTc sestamibi scan showed no changes, suggesting ectopic production of PTH. Being a rare event, malignant hypercalcemia from intact PTH ectopic production should be considered in these patients.

## Introduction

Malignant hypercalcemia is relatively common in a wide range of cancers, from solid tumors to malignant hematologic tumors. This event may occur due to mechanisms like the presence of osteolytic metastases with local release of cytokines, tumor production of calcitriol, and tumor secretion of parathyroid hormone-related protein (PTH-rP), which is a humoral factor synthesized and secreted by tumor cells that exert similar biochemical changes as those observed in primary hyperparathyroidism [1,2]. However, there are some reports of non-parathyroid tumors producing intact parathyroid hormone (PTH) [3-9]. Herein, we report a case of hypercalcemia with hyperparathyroidism from ectopic production of intact PTH.

## Case presentation

An 84-year-old male patient was admitted to the hospital with a non-productive cough and anorexia for one month, as well as constipation for three days and a single episode of small-volume hemoptysis. He was hemodynamically stable and had no nausea or vomiting, polyuria, weight loss, night sweats, dyspnea, palpitations, or chest pain. This patient had smoked for six pack-years and had no drinking history. He had no family history of cancer. 

Prior medical history included heart failure with preserved left ejection fraction, essential arterial hypertension (treated with amlodipine, bisoprolol, and perindopril), dyslipidemia (treated with atorvastatin and ezetimibe), obstructive sleep apnea syndrome (under nocturnal continuous positive airway pressure therapy), and rheumatic polymyalgia (treated with deflazacort). 

**Figure 1:**
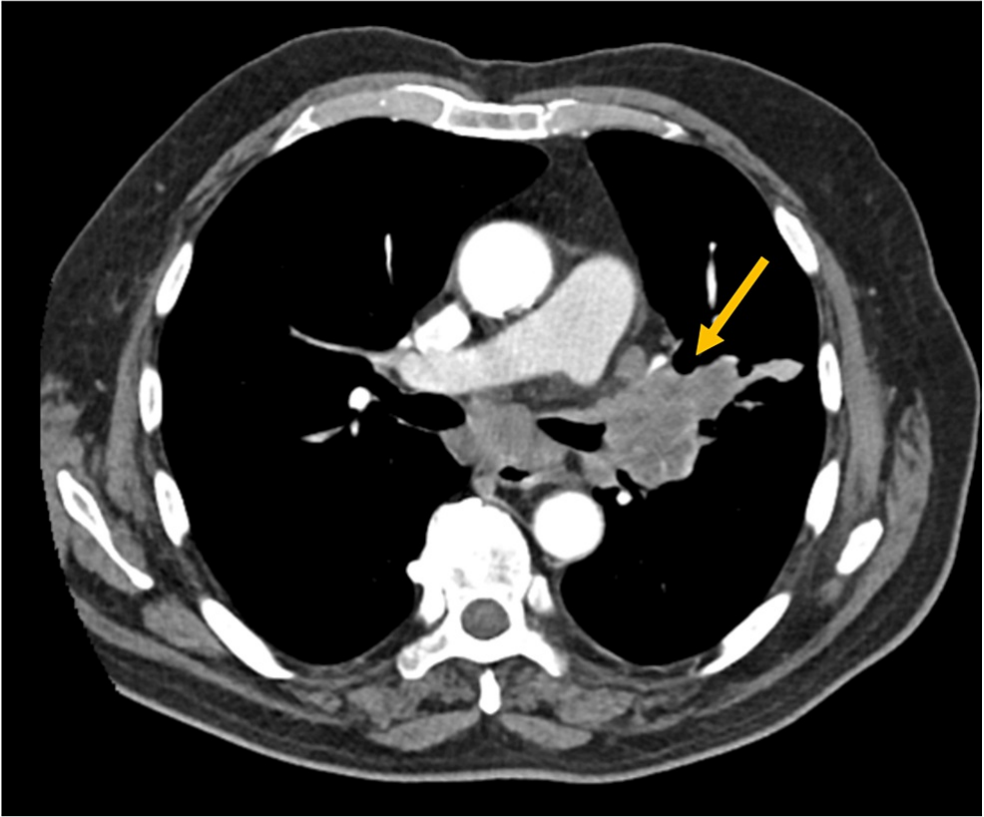
Thoracic CT scan showing a pulmonary mass invading the left pulmonary artery and vein

A thoracic and abdominal CT angiography showed a left lung mass on the hilum with invasion of the pulmonary artery and vein as well as the lingula bronchus, with no evidence of active bleeding (Figure 1).

**Figure 2:**
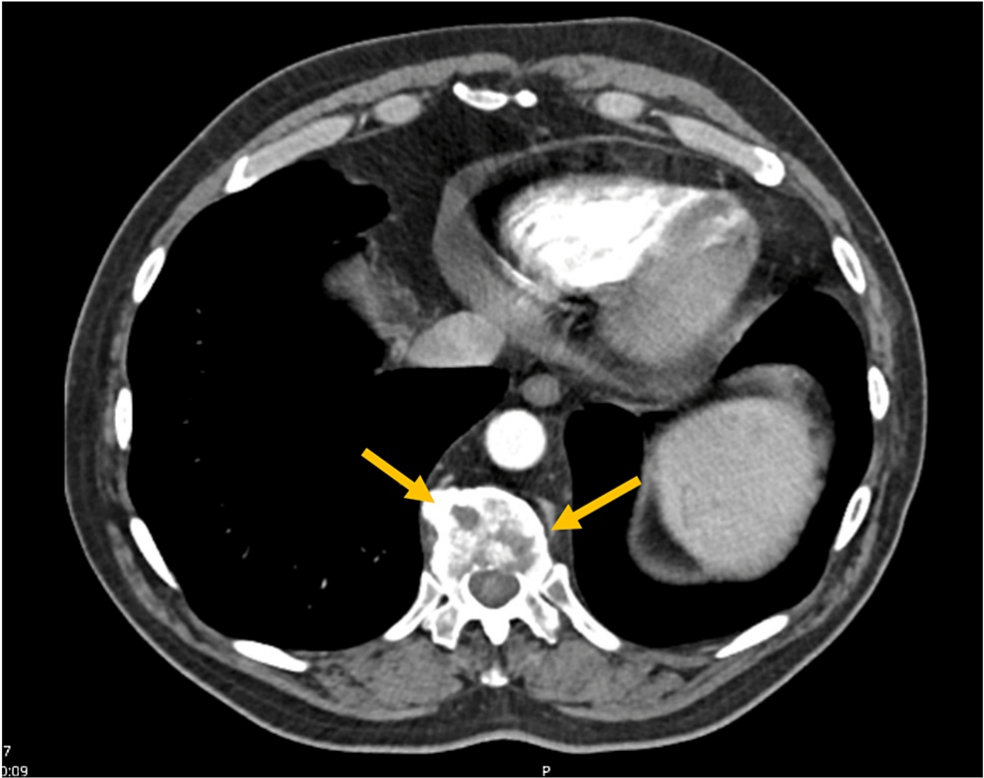
Thoracic CT scan showing vertebral lytic lesions

Multiple adenopathies were also described on the mediastinum, as well as two hepatic nodules suggesting secondary lesions. There were also lytic lesions in the pelvis, sacrum, and in multiple lumbar and dorsal vertebrae (Figure 2).

Laboratory tests were remarkable for an elevated ionized calcium value of 4.02 mEq/L (reference range: 2.26-2.64 mEq/L) and serum phosphorous of 2.7 mg/dL (reference range: 2.7-4.5 mg/dL) without renal failure (Table 1). His electrocardiogram was unremarkable. He received fluid therapy, diuretics, and zoledronic acid, with a subsequent decrease in calcium serum levels. 

**Table 1:** Laboratory data

Variable	Reference Range	On admission
Hemoglobin (g/dL)	13-18	13.4
Leucocytes (x10^9^/L)	4-11	4.73
Platelets (x10^9^/L)	150-400	127
Alanine aminotransferase (U/L)	10-37	46
Aspartate aminotransferase (U/L)	10-37	21
Gamma-glutamyltransferase (U/L)	10-49	60
Alkaline phosphatase (U/L)	30-120	107
Total bilirubin (mg/dL)	<1.20	0.7
Direct bilirubin (mg/dL)	<0.4	0.14
Albumin (g/dL)	38-51	35
Sodium (mmol/L)	135-147	137
Potassium (mmol/L)	3.5-5.1	4.2
Ionized Calcium (mmol/L)	2.26-2.64	4.02
Magnesium (mmol/L	1.55-2.05	1.33
Phosphorus (mg/dL)	2.7-4.5	2.7
Urea nitrogen (mg/dL)	10-50	57
Creatinine (mg/dL)	0.67-1.17	0.98
Glucose (mg/dL)	75-110	106
Lactate dehydrogenase (U/L)	135-225	705
Parathyroid hormone (pg/mL)	10-65	117
Vitamin D (ng/mL)	>20	24
Urinary calcium /24h (mmol/L)	5-15	9.1

In order to study the etiology of this hypercalcemia, serum PTH measurement was made showing a level of 117pg/mL (reference range: 10-65 pg/mL), suggesting primary hyperparathyroidism. PTH-rP measurement was not available at our laboratory. The 24-hour urinary calcium excretion and serum vitamin D were within the normal range. A parotid standard technetium (99mTc) sestamibi scan showed no changes on the parathyroid glands, suggesting ectopic production of intact PTH as an additional mechanism to hypercalcemia. 

A lung biopsy was performed, and histology showed a small cell lung cancer with the perinuclear expression of CK8/l8 and strong expression of synaptophysin and thyroid transcription factor 1 (TTF-1). This tumor was classified as stage T4N3M1c (staged by CT scan) [10]. Palliative chemotherapy was initiated with etoposide and carboplatin with progressive lowering of serum ionized calcium levels.

## Discussion

The ectopic expression of hormones by cancer cells has been recognized as a cause of many paraneoplastic disorders, including Cushing syndrome, syndrome of inappropriate secretion of antidiuretic hormone (SIADH), and hypercalcemia [5,11]. Malignant hypercalcemia is most frequently associated with the production of PTH-rP. However, some reports show ectopic production of intact PTH by non-parathyroid tumors. These involve small cell carcinoma of the lung [12], squamous cell carcinoma [13], ovarian carcinoma [5], thymoma [3], neuroectodermal tumor [6], papillary thyroid carcinoma [7], bladder carcinoma [4], rhabdomyosarcoma [14], pancreatic cancer [15], and lymphoma [8].

The mechanism underlying these non-suppressible high serum PTH levels are not clearly known. Suggested mechanisms include calcium-independent PTH secretion, altered calcium set-point that need higher serum calcium to suppress PTH secretion, and decreased PTH clearance [2,16]. However, concomitant primary hyperparathyroidism should be considered in these situations [17]. 

In a study of ectopic PTH production in a patient with ovarian carcinoma and hypercalcemia, genomic analysis of tumor DNA suggested the possibility of the existence of DNA amplification and rearrangements uncovering an enhancer sequence or deleting a suppressor sequence to account for high PTH mRNA expression, leading to PTH overexpression [5]. This way, the development of hypercalcemia with high intact serum PTH levels in a patient with malignancy may suggest new changes of the PTH gene or activation of suppressor/enhancement genes in these cells [14,15]. 

In our patient, the presence of osteolytic lesions as well as a neoplastic process (through PTH-rP) could explain the hypercalcemia. However, PTH serum levels were elevated, supporting some contribution of intact production of PTH regardless of serum calcium levels.

The 24-hour urinary calcium excretion as well as vitamin D levels should be evaluated in these patients. In the present case, vitamin D levels and 24-hour urinary calcium excretion were within normal limits. A parathyroid 99mTc sestamibi scan showed no changes, supporting the possibility of ectopic production of intact PTH by the tumor. 

Fluid therapy, diuretics, and zoledronic acid will only lower the serum calcium temporarily. To treat hypercalcemia due to ectopic production of PTH, its underlying cause should be addressed through the reduction of the tumoral activity such as chemo or radiotherapy, surgery removal, or other directed therapies [18]. For our patient, we decided to start palliative chemotherapy, which had a calcium-lowering effect.

## Conclusions

Hypercalcemia is a common complication of malignancy caused by heterogenous groups of tumor-derived factors that disrupt normal calcium homeostasis. Even if it is a rare finding, intact PTH ectopic production should be suspected in patients with a known malignancy along with elevated serum calcium and PTH without abnormally active parathyroid glands. Prompt therapy should be administered in order to reduce tumoral activity and prevent life-threatening features of hypercalcemia. 

## References

[1] Clines GA (2011). Mechanisms and treatment of hypercalcemia of malignancy. Curr Opin Endocrinol Diabetes Obes.

[2] Sriussadaporn S, Phoojaroenchanachai M, Ploybutr S (2007). Hypercalcemia of malignancy: a study of clinical features and relationships among circulating levels of calcium, parathyroid hormone and parathyroid hormone-related peptide. J Med Assoc Thail.

[3] Rizzoli R, Pache JC, Didierjean L, Burger A, Bonjour JP (1994). A thymoma as a cause of true ectopic hyperparathyroidism. J Clin Endocrinol Metab.

[4] Eid W, Wheeler TM, Sharma MD (2004). Recurrent hypercalcemia due to ectopic production of parathyroid hormone-related protein and intact parathyroid hormone in a single patient with multiple malignancies. Endocr Pract.

[5] Nussbaum SR, Gaz RD, Arnold A (1990). Hypercalcemia and ectopic secretion of parathyroid hormone by an ovarian carcinoma with rearrangement of the gene for parathyroid hormone. N Engl J Med.

[6] Strewler GJ, Budayr AA, Clark OH, Nissenson RA (1993). Production of parathyroid hormone by a malignant nonparathyroid tumor in a hypercalcemic patient. J Clin Endocrinol Metab.

[7] Iguchi H, Miyagi C, Tomita K, Kawauchi S, Nozuka Y, Tsuneyoshi M, Wakasugi H (1998). Hypercalcemia caused by ectopic production of parathyroid hormone in a patient with papillary adenocarcinoma of the thyroid gland. J Clin Endocrinol Metab.

[8] Goldman JW, Becker FO (1978). Ectopic parathyroid hormone syndrome. Occurrence in a case undifferentiated lymphoma with bone marrow involvement. Arch Intern Med.

[9] Simpson EL, Mundy GR, D'Souza SM, Ibbotson KJ, Bockman R, Jacobs JW (1983). Absence of parathyroid hormone messenger RNA in nonparathyroid tumors associated with hypercalcemia. N Engl J Med.

[10] Goldstraw P, Chansky K, Crowley J (2016). The IASLC lung cancer staging project: proposals for revision of the TNM stage groupings in the forthcoming (eighth) edition of the TNM classification for lung cancer. J Thorac Oncol.

[11] Anwar A, Jafri F, Ashraf S, Jafri MA, Fanucchi M (2019). Paraneoplastic syndromes in lung cancer and their management. Ann Transl Med.

[12] Yoshimoto K, Yamasaki R, Sakai H (1989). Ectopic production of parathyroid hormone by small cell lung cancer in a patient with hypercalcemia. J Clin Endocrinol Metab.

[13] Nielsen PK, Rasmussen AK, Feldt-Rasmussen U, Brandt M, Christensen L, Olgaard K (1996). Ectopic production of intact parathyroid hormone by a squamous cell lung carcinoma in vivo and in vitro. J Clin Endocrinol Metab.

[14] Wong K, Tsuda S, Mukai R, Sumida K, Arakaki R (2005). Parathyroid hormone expression in a patient with metastatic nasopharyngeal rhabdomyosarcoma and hypercalcemia. Endocrine.

[15] VanHouten JN, Yu N, Rimm D, Dotto J, Arnold A, Wysolmerski JJ, Udelsman R (2006). Hypercalcemia of malignancy due to ectopic transactivation of the parathyroid hormone gene. J Clin Endocrinol Metab.

[16] Uchida K, Tanaka Y, Ichikawa H (2017). An excess of CYP24A1, lack of CaSR, and a novel lncRNA near the PTH gene characterize an Ectopic PTH-producing tumor. J Endocr Soc.

[17] Basso U, Maruzzo M, Roma A, Camozzi V, Luisetto G, Lumachi F (2011). Malignant hypercalcemia. Curr Med Chem.

[18] Skrabanek P, McPartlin J, Powell D (1980). Tumor hypercalcemia and "ectopic hyperparathyroidism". Medicine (Baltimore).

